# Investigating the beneficial effects of a WO_3_ seed layer on the mechanical and photoelectrochemical stability of WO_3_|BiVO_4_|NiFeOOH photoanodes under operational conditions

**DOI:** 10.1557/s43579-025-00788-9

**Published:** 2025-08-18

**Authors:** George H. Creasey, Andreas Kafizas, Anna Hankin

**Affiliations:** 1 Department of Chemical Engineering, Imperial College London, South Kensington, London, SW7 2AZ UK; 2 Department of Chemistry, The Molecular Science Research Hub, Imperial College, White City, London, W12 0BZ UK

**Keywords:** Adhesion, Bi, Chemical vapor deposition (CVD), Devices, Durability, Heterostructure, Hybrid, Kinetics, Morphology, Nanostructure, Oxidation, Oxide, Photoelectrochemical, Renewable, Semiconducting, V

## Abstract

Scalable and durable photoelectrodes are essential for technological breakthroughs in photoelectrochemical systems, yet the fragility of nanostructured photocatalyst materials in industrially relevant operating conditions is rarely explored. Herein, we advance understanding of the importance of morphology and temperature on stability and performance of nanostructured WO_3_|BiVO_4_|NiFeOOH photoanodes. The integration of a planar WO_3_ seed layer beneath nanostructured WO_3_, improved mechanical stability at 40°C with flowing electrolyte approximately twofold compared with materials where a seed layer was not integrated. This work provides a pathway through which robust photoelectrode systems can be engineered to enable the advancement of up-scaled photoelectrochemical water splitting.

**Graphical abstract:**

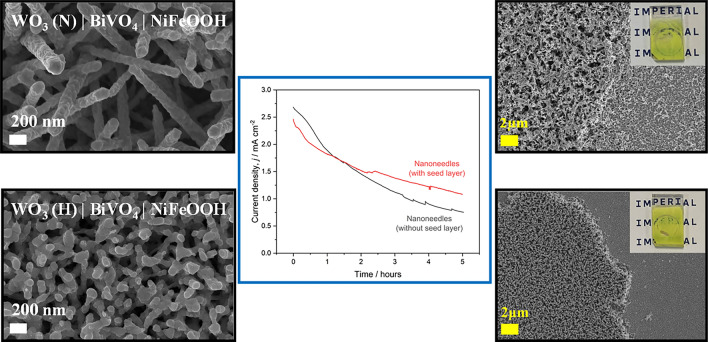

**Supplementary Information:**

The online version contains supplementary material available at 10.1557/s43579-025-00788-9.

## Introduction

Photoelectrodes are key components of photoelectrochemical reactors—devices used for synthesizing ‘solar fuels’ and ‘solar chemicals’.^[1]^ The purpose of a photoelectrode is to: (i) absorb solar photons and generate electron–hole pairs in the semiconducting material layer(s) and (ii) enable the transfer of these electrons/holes *via* (photo) electrochemical reduction/oxidation. Reactions of interest include water splitting into hydrogen and oxygen, as well as more complex processes such as CO_2_ reduction and biomass or organic pollutant oxidation.

Photoelectrodes are often developed to have nanotextured surfaces for enhanced light absorption, charge separation and charge transfer.^[2–5]^ Performance improvements are typically assessed through measuring electrical currents during potentiodynamic scans and chronoamperometric tests. However, characterization of photoelectrode materials is mostly limited to well-controlled lab-based experiments, which are not representative of real outdoor operating conditions.

For commercially viable photoelectrochemical systems, photoelectrodes must be designed with consideration for scalability and device integration. Using scalable photoelectrode synthesis routes, such as chemical vapor deposition (CVD) or electrodeposition, can ensure that homogeneous photocatalyst layers can be translated from small-scale (< 1 cm^2^) to medium and large-scale prototype devices. To integrate with these devices, photoelectrodes must also be designed to withstand more dynamic operating conditions than those they are usually subjected to in a controlled laboratory environment. Devices will be designed to enable photoelectrode replacement and prolong device lifetimes, but downtime for component replacement should be minimized in a long-term operation strategy to maximize production rates.

When photoelectrochemical reactors are eventually deployed industrially, they will operate with a continuously flowing electrolyte that will simultaneously deliver the reactant to the photoactive surface, remove the product for harvesting and enable heat management. This is the case in the largest photocatalytic water splitting installation to date (≈ 100 m^2^), which utilizes catalyst sheets.^[6]^ Furthermore, the irradiance at a given location will vary as a function of time of year and time of day and will fluctuate due to changes in cloud cover. The electrolyte temperature will most likely be affected by irradiance and wind speed if the reservoirs and flow circuit are above ground, and hence will also change over the course of each day. Hence, there will be significant departure from the idealized conditions of the lab, where irradiance is usually constant at AM 1.5G, the temperature is in the range ≈20–25°C and the electrolyte is quiescent.

**Figure 1:**
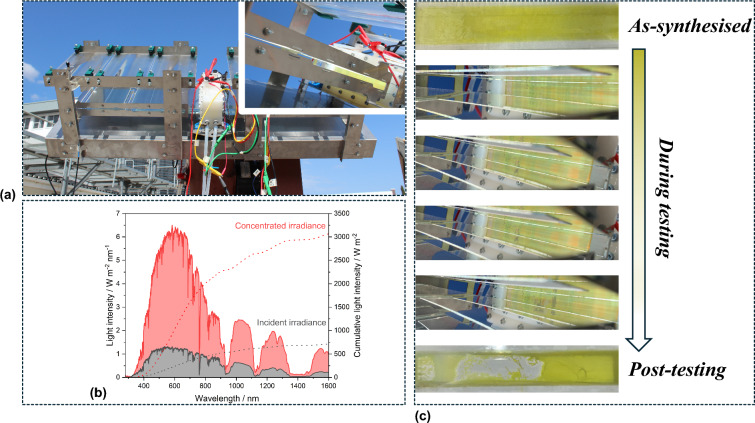
(a) Photographs of the up-scaled photoelectrochemical reactor, coupled with concentrating optics, which was tested in South Africa under concentrated natural sunlight. (b) Representative measured irradiance during water splitting experiments with concentrating optics. (c) Observed degradation of the nanostructured WO_3_ | BiVO_4_ | NiFeOOH at different stages of testing, from a pristine electrode to post- 2 h experiment.

We observed the detrimental influence of real operating conditions on photoelectrode performance and longevity during field tests carried out at Stellenbosch University, South Africa, between March and May 2024. Our reactor utilized 15 cm × 2 cm FTO | WO_3_ | BiVO_4_ | NiFeOOH heterojunction photoanodes with nanoneedle morphology, synthesized *via* aerosol assisted chemical vapor deposition (AA-CVD). The reactor was mounted on a 2-axis tracking platform, shown in Fig. 1. The reactor was operated in two optical configurations for redirecting the incident radiation laterally into the reactor in which the (photo)electrodes were oriented vertically: (i) linear Fresnel lenses coupled with stepped Al waveguides for delivering concentrated light and (ii) mirrors (Al-coated mirror for irradiating the photoanode). Continuously flowing electrolyte was employed in all experiments. The electrolyte reservoir temperature was recorded to increase up to 40°C, depending on the reactor irradiation mode and ambient temperature on the day. Photoelectrode degradation was observed to occur the fastest at highest irradiance (with concentrated light) and on hottest days. It was not clear whether the degradation was induced predominantly by (photo)electrochemical bismuth oxidation, known to occur to some extent in all the commonly used electrolytes,^[7,8]^ or by the delamination of the nanostructured WO_3_ from the FTO^[9]^ (WO_3_ was grown in the form of nanoneedles, which were subsequently coated with BiVO_4_). We believed that heat caused the delamination and that this was exacerbated by electrolyte flow; hence, we investigated this subsequently in greater detail and present the results herein.

We corroborated the inverse correlation between nanostructure and mechanical stability in FTO | WO_3_ | BiVO_4_ heterojunction photoelectrodes, synthesized *via* AA-CVD.^[10]^ While a WO_3_ nanoneedle layer coated with BiVO_4_ was found to deliver a superior photoelectrochemical performance in quiescent conditions, it delaminated under electrolyte flow during longer-term operation at 20°C. Planar WO_3_ has excellent mechanical stability but was previously ruled out for further investigation due to its very poor photoelectrochemical performance.^[10]^ In this study, we integrate a thin planar seed layer between FTO and nanostructured WO_3_ with the objective to provide a greater degree of adhesion at the FTO | WO_3_ interface, while maintaining the advantageous properties of nanostructuring.

Herein we present efforts to develop further understanding of the effects of morphology and temperature on the stability of WO_3_ | BiVO_4_ | NiFeOOH electrodes synthesized by AA-CVD. While the majority of studies focus on delivering the highest possible water oxidation performances in highly controlled systems, we investigate operating conditions that are more representative of future up-scaled photoelectrochemical devices. Addressing future engineering challenges in the laboratory is imperative to ensuring viable photoelectrochemical systems in which materials and devices have high compatibility, performance and longevity.

## Materials and methods

### Photoelectrode synthesis

Photoelectrodes were synthesized by the sequential AA-CVD of WO_3_, BiVO_4_ and NiFeOOH on TEC-15 FTO glass substrates, in a purpose-built reactor (shown in Fig. S1). The AA-CVD deposition methods were adapted from previous work by the authors.^[10,11]^ Before the deposition of WO_3_, it was first necessary to prepare and pre-treat the FTO glass substrates, which were cut into 1.5 cm × 2.0 cm pieces. The pre-treatment comprised sequential ultrasonic cleaning in detergent water, deionized water, acetone and isopropanol, each for 10 min. The substrates were then dried with compressed nitrogen to remove any residual solvent or debris.

#### Seed (planar) and nanoneedle WO_3_ layer morphology synthesis

For the deposition of WO_3_, a precursor solution was prepared containing 0.16 g (11.4 mM) of tungsten hexacarbonyl (Merck,  ≥ 97%) fully dissolved in 26.7 mL acetone and 13.3 mL methanol. The FTO substrates were placed on a heated carbon block in the CVD reactor. The precursor was aerosolized by an ultrasonic humidifier (2 MHz, Liquifog, Johnson Matthey) and carried through the CVD reactor by compressed nitrogen. To prepare electrodes with a planar “seed” layer, 10 mL of precursor was passed through the reactor with a nitrogen carrier gas flowrate of 1.5 L min^−1^, with a temperature setpoint of 325°C. To grow WO_3_ nanoneedles on the seed layer, the temperature was then increased to 375°C and the remaining 30 mL of precursor were passed through the CVD chamber with a nitrogen carrier gas flowrate of 2.0 L min^−1^. A hybrid WO_3_ morphology was grown with a reaction temperature of 350°C and a nitrogen carrier gas flowrate of 1.5 L min^−1^. To grow WO_3_ films without a seed layer, 40 mL of precursor was passed through the reactor. The hybrid and nanoneedle WO_3_ films were grown with reaction temperatures of 350°C and 375°C, and carrier gas flowrates of 1.5 L min^−1^ and 2.0 L min^−1^, respectively. After the deposition of WO_3_, the samples were placed in a muffle furnace and annealed in air for 2 h at 500°C (ramp rate 200°C h^−1^).

#### BiVO_4_ photoabsorbing layer synthesis

In the second step, BiVO_4_ films were deposited onto WO_3_ to form the WO_3_ | BiVO_4_ heterojunction. For the deposition of BiVO_4_, a precursor solution containing 0.0697 g (5 mM) of vanadium (III) acetylacetonate (Merck, ≥ 97%) and 0.0881 g (5 mM) of triphenyl bismuth (Alfa Aesar, ≥ 99%) fully dissolved in 15 mL acetone and 5 mL methanol was prepared. Similarly to the previous deposition, the substrates already coated in WO_3_ were placed on a graphitic carbon block, which in this case was heated to 400°C for the synthesis of all BiVO_4_ films. All 20 mL of the precursor were aerosolized and passed through the CVD reactor, carried by 1 L min^−1^ compressed air. After the deposition of BiVO_4_, the samples were placed in a muffle furnace and annealed in air for 2 h at 500°C (ramp rate 200°C h^−1^).

#### NiFeOOH co-catalyst deposition

In the third and final step, NiFeOOH films were deposited onto the WO_3_ | BiVO_4_ heterojunction. For the deposition of NiFeOOH, a precursor solution containing 20.55 mg (4 mM) of nickel (II) acetylacetonate (Merck, ≥ 97%) and 7.06 mg (1 mM) of iron (III) acetylacetonate (Merck,  ≥ 97%) fully dissolved in 13.3 mL acetone and 6.7 mL methanol was prepared. Similarly to the previous depositions, the substrates already coated in WO_3_ | BiVO_4_ were placed on a graphitic carbon block, which in this case was heated to 160°C for the synthesis of all NiFeOOH films. All 20 mL of the precursor were aerosolized and passed through the CVD reactor, carried by 1.5 L min^−1^ compressed nitrogen. After the deposition of NiFeOOH, the samples were placed in a muffle furnace and annealed in air for 2 h at 160°C (ramp rate 100°C h^−1^).

### In situ photoelectrochemical performance

As-synthesized photoanode electrodes were characterized photoelectrochemically. Photoanodes were back-illuminated in a ‘PTFE Photoelectrochemical Flow H-Cell’ from Redox.me. The cell was equipped with Redox.me Pt coil and refillable AgCl|Ag (sat. KCl, 0.197 V_SHE_ at 25°C) auxiliary electrodes. Electrolyte flow was regulated by a Redox.me peristaltic pump. The temperature of the electrolyte was controlled by placing the electrolyte reservoir on a AREC 7 CONNECT digital hot plate stirrer with ceramic top, equipped with a PT100 probe and clamp (VELP Scientifica Srl). Photoelectrochemical measurements were conducted in 1 M borate buffer (1 M H_3_BO_3_,  ~ 20 g L^−1^ NaOH titrated to pH 9). Electrolytes were prepared with analytical reagent standard Milli-Q-water (Millipore Corp., 18.2 MΩ·cm at 25°C).

The photoelectrochemical cell was irradiated by a Sun2000 Solar Simulator (Class A, Abet Technologies) equipped with a 550 W Xe lamp and AM 1.5G filter. The working distance was adjusted to ensure an equivalent cumulative incident light intensity to the reactor of one sun (100 mW cm^−2^). The light spectrum and intensity was characterized by StellarNet Black Comet UV/vis (280–900 nm) and Dwarf Star (900–1700 nm) spectrophotometers, interfaced with StellarPro software. A representative light spectrum, compared with the NREL AM 1.5G standard, is provided in Fig. S2.

The photoelectrochemical performance was characterized by linear sweep voltammetry (LSV) and chronoamperometry (CA). Photoelectrochemical measurements were conducted in accordance with established protocols for photoelectrochemical water splitting.^[12,13]^ LSVs were typically conducted from the open circuit potential (under continuous illumination) to 1.7 V_RHE_, with a scan rate of 10 mV s^−1^. Three linear sweep voltammograms were measured consecutively on each sample under continuous illumination and results from the third scan were used in the analysis. This was followed by a scan using chopped light, with a chopping frequency of 1 Hz. Photoanode stability was quantified by recording the photocurrent density as a function of time during chronoamperometry at 1.23 V_RHE_. LSV and CA measurements were conducted either at room temperature or with electrolyte temperatures of 40°C. The charge separation and injection efficiencies were determined through LSV tests in 1 M sodium borate buffer (pH 9), with and without 0.5 M Na_2_SO_3_ sacrificial reagent. Charge transfer and injection efficiencies were then calculated by the following equations:

1 $$j_{{{\text{photo}}}}^{{{\text{H}}_{2} {\text{O}}}} = j_{{{\text{abs}}}} \times \phi_{{{\text{sep}}}} \times \phi_{{{\text{inj}}}}$$

2 $$j_{{{\text{photo}}}}^{{{\text{Na}}_{2} {\text{SO}}_{3} }} = j_{{{\text{abs}}}} \times \phi_{{{\text{sep}}}}$$

where ${j}_{\text{photo}}^{{\text{H}}_{2}\text{O}}$, ${j}_{\text{photo}}^{{\text{Na}}_{2}{\text{SO}}_{3}}$ are the measured current densities for H_2_O and Na_2_SO_3_ oxidation, ${j}_{\text{abs}}$ is the theoretical photocurrent density generated when 100% of solar energy (AM 1.5 G at 1 sun power) absorbed by the material is converted into photocurrent, ${\phi }_{\text{sep}}$ is the charge separation efficiency, ${\phi }_{\text{inj}}$ is the charge injection efficiency.

All electrode potentials are reported versus the reversible hydrogen electrode (V_RHE_ in volts), calculated using the Nernst equation:

3 $${\text{V}}_{{{\text{RHE}}}} = {\text{V}}_{{{\text{AgCl}}|{\text{Ag}}}} + \left( {0.0591 \times pH} \right) + {\text{V}}_{{{\text{AgCl}}|{\text{Ag}}}}^{\theta }$$

where ${V}_{\text{AgCl}|\text{Ag}}$ is the electrode potential vs the AgCl|Ag reference electrode and ${V}_{\text{AgCl}|\text{Ag}}^{\theta }$ is the standard potential of the reference electrode (all in volts).

### Ex situ photoelectrode characterization

The crystal structure of the photoanodes was characterized by X-ray diffraction using a Bruker D2 Phaser diffractometer with parallel beam optics equipped with a Lynx-Eye detector. X-rays were generated using a Cu source (*V* = 30 kV, *I* = 10 mA) with Cu K-alpha radiation (*λ*_1_ = 1.54056 Å, *λ*_2_ = 1.54439 Å) emitted with an intensity ratio of 2:1. The diffraction patterns were collected at scattering angles between 17° ≤ 2θ ≤ 53°, with a step size of 0.02° s^−1^.

Raman spectra were obtained using a Bruker SENTERRA II Raman Microscope. Samples were excited with a 532 nm laser of 6.25 mW power over a 50 μm diameter irradiation area. Spectra were recorded with Raman shifts from 50 to 1000 cm^−1^ with a resolution of 1.5 cm^−1^.

Surface morphologies were characterized by scanning electron microscopy using a Zeiss Gemini Sigma300 FEG SEM or a Zeiss Auriga Cross Beam FIB-SEM, with EHT of 5 keV and InLens detector. The samples were characterized with magnifications up to 100000 times, providing resolution to the order of 10 s of nanometres. A typical working distance of 4 to 5 mm was used.

The surface compositions of the photoanodes were also characterized by X-ray photoelectron spectroscopy to determine the oxidation states of constituent elements using a Thermo Fisher K-Alpha Plus XPS spectrometer, equipped with a monochromated Al K-alpha X-ray source. Surface charge was compensated using a flood gun and binding energies were referenced to adventitious carbon peaks seen in the C 1 s binding energy region. XPS analysis was carried out using CasaXPS.

Optical properties of the photoanodes were characterized by UV–vis spectroscopy, using a Shimadzu UV-2600 UV–vis spectrometer. Transmittance and reflectance of the samples were measured between 270 and 800 nm, with the absorptance calculated as shown in Equation (4).

4 $${\text{Absorptance}} \left( \% \right) = 100 - {\text{Transmittance}} \left( \% \right) - {\text{Reflectance}} \left( \% \right)$$

The optical bandgaps were obtained graphically using Tauc plots, as described by the authors in previous work.^[10]^

## Results

To investigate the effects of temperature and photoanode morphology, a range of nanostructured and planar WO_3_ | BiVO_4_ | NiFeOOH films were prepared and tested with ambient and elevated (40°C) electrolyte temperatures. A scheme summarizing the synthesis conditions and photographs of the photoanodes tested herein is presented in Table S1.

### Physical materials characterization of WO_3_ | BiVO_4_ | NiFeOOH photoanodes

**Figure 2:**
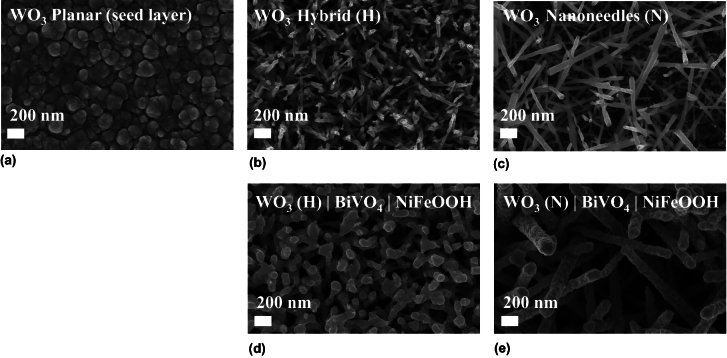
SEM images at 100000 times magnification. (a) Planar (‘P’) WO_3_ synthesized at 325°C; (b) hybrid (‘H’) WO_3_ synthesized at 350°C; (c) WO_3_ nanoneedles (‘N’) synthesized at 375°C; (d) WO_3_ (H) | BiVO_4_ | NiFeOOH; (e) WO_3_ (‘N’) | BiVO_4_ | NiFeOOH.

As reported previously, the planar WO_3_ films appeared very transparent in contrast to the hybrid and nanoneedle films, which were the most opaque.^[9,10]^ Whereas the nanostructured films had thicknesses between 400 and 500 nm, the planar films were much thinner, at approximately 100 nm thickness.^[10]^ SEM images of the three WO_3_ morphologies are shown in Fig. 2. The planar films grown at 325°C had a very high level of contact with the FTO compared with the nanostructured films, enabling better adhesion. The films grown at 375°C were more complex nanostructures with a reduced degree of contact with poorer adhesion. “Hybrid” WO_3_, grown at the intermediate temperature of 350°C grew with greater adhesion to the FTO than the nanoneedles, while also having a much higher (photo) electroactive surface area than the planar films. SEM images of the photoanodes following the deposition of BiVO_4_ showed the conformal growth of BiVO_4_ on WO_3_ forming nanoporous WO_3_ | BiVO_4_ photoanodes. As shown in previous work, the deposition of NiFeOOH, with thickness of approximately 5 nm, was not visible by SEM.^[10]^

WO_3_ morphologies investigated in this study, and the as-synthesized WO_3_ | BiVO_4_ | NiFeOOH photoanodes using either hybrid WO_3_ (H) or WO_3_ nanoneedles (N).

**Figure 3:**
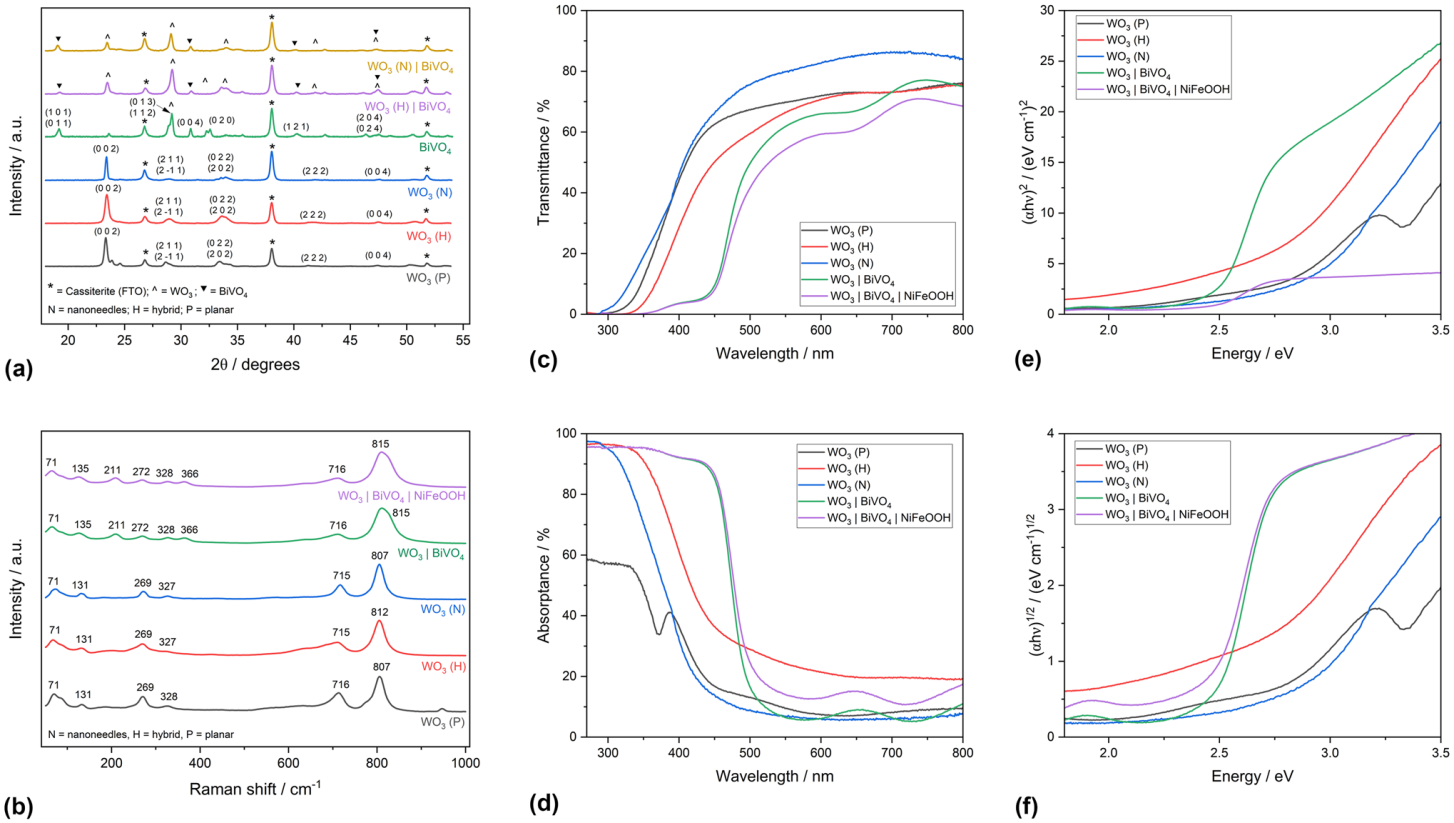
Physical materials characterization of the WO_3_ | BiVO_4_ | NiFeOOH photoanodes. (a) X-ray diffraction patterns; (b) Raman spectra; (c) UV–vis transmittance; (d) UV–vis absorptance; (e, f) Tauc plots.

XRD and Raman analysis [shown in Fig. 3(a, b)] showed that all WO_3_ and BiVO_4_ films were phase pure and monoclinic (γ-WO_3_ and m-BiVO_4_). Although the XRD patterns of the different WO_3_ morphologies were broadly the same, there were some differences including additional peaks for the planar films around *2θ* = 21°, corresponding to the Miller indices (0 2 0) and (2 0 0). These are characteristic of planar structured WO_3_ films and are in line with previous reports.^[9,10,14]^ There was also some variation in the bond vibrations in Raman with different WO_3_ morphologies, but all three morphologies included the prominent bands at 807 cm^−1^ and 716 cm^−1^, corresponding to O–W–O and W_2_O_6_ bond vibrations, as reported previously by the authors.^[10]^ The optical properties of the samples can be compared in [Fig. 3(c–f)]. The WO_3_ hybrid structure possessed the narrowest bandgap of the three WO_3_ morphologies, at 2.69 eV (~ 461 nm). When conformally coated in BiVO_4_ and NiFeOOH, the resulting WO_3_ | BiVO_4_ | NiFeOOH photoanode possessed a bandgap of 2.47 eV (~ 502 nm), with these values in line with previous reports.^[10,15,16]^ XPS analysis of the W 4f, Bi 4f and V 2p environments (shown in Fig. S3) provided evidence that tungsten, bismuth and vanadium were in their expected oxidation states of + 6, + 3 and + 5, respectively. Additionally, analysis of the Ni 2p and Fe 2p environments (shown in Fig. S4) provided evidence of the successful loading of NiFeOOH.

### Photoelectrochemical testing of WO_3_|BiVO_4_|NiFeOOH photoanodes

**Figure 4:**
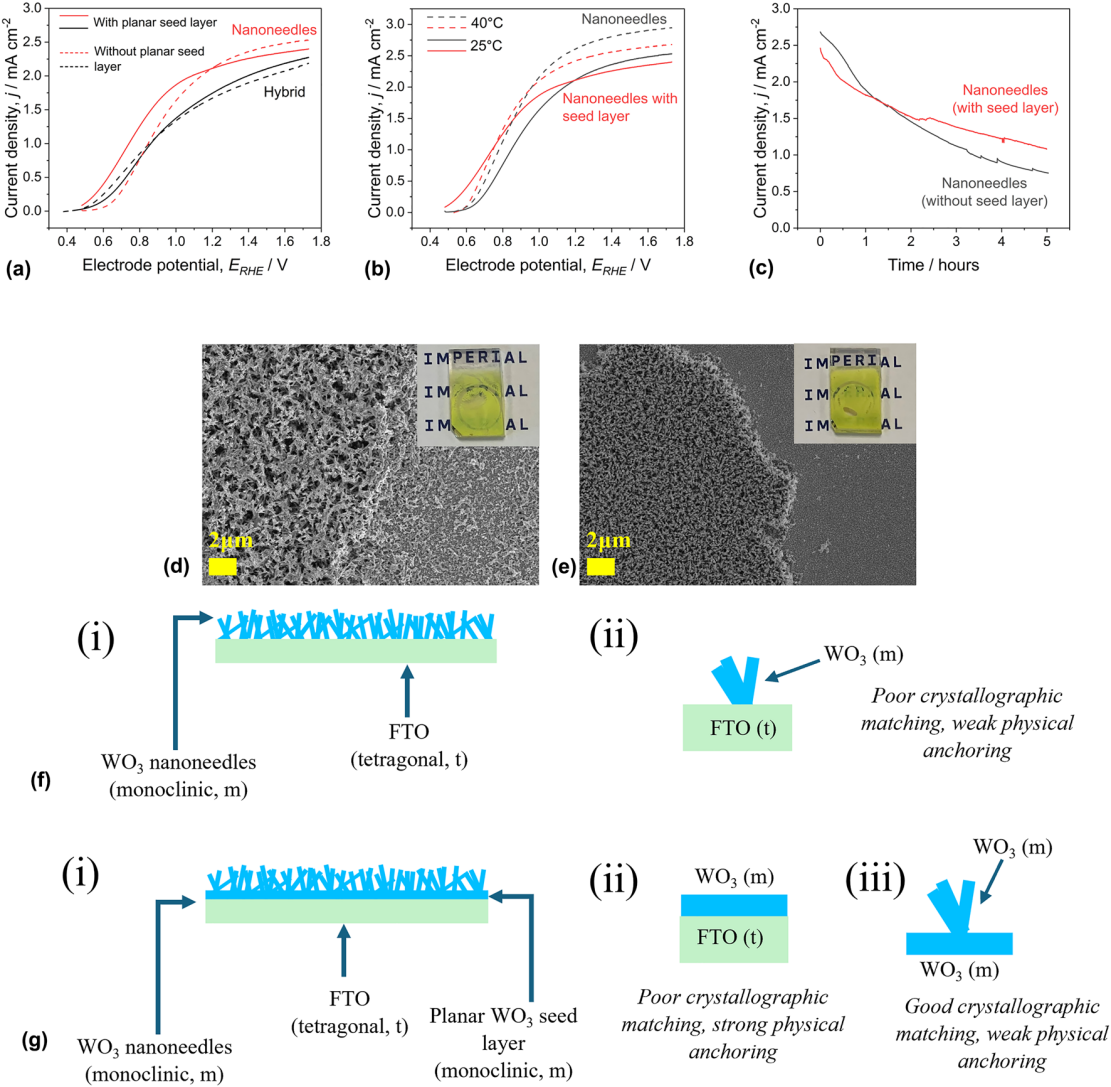
Photoelectrochemical characterization, post hoc microscopy and physical bonding of WO_3_ | BiVO_4_ | NiFeOOH photoanodes, with and without planar WO_3_ seed layers. During all PEC measurements, the cell was irradiated by one sun irradiance (AM 1.5 G, 100 mW cm^−2^). LSV tests were conducted with scan rates of 10 mV s^−1^. All PEC measurements were taken with a 1 M borate buffer electrolyte (pH 9), with a flowrate of 100 mL min^−1^ (1.25 cm s^−1^). (a) LSV tests of nanostructured electrodes with and without seed layers at room temperature. (b) LSV tests of nanostructured electrodes with planar seed layers, at electrolyte temperatures of 25°C and 40°C. (c) Photocurrent density of nanostructured electrodes, with and without seed layers during 5-h chronoamperometry stability testing at 1.23 V_RHE_ under flow in 40°C electrolyte. SEM images at 10000 times magnification after the 5-h stability tests for electrodes without (d) and with (e) planar seed layers. Physical bonding of the electrodes without (f) and with (g) planar seed layers.

The effect of the seed layer on water oxidation performance was initially assessed by comparing the nanostructured WO_3_ | BiVO_4_ | NiFeOOH photoanodes during linear sweep voltammetry and chronoamperometry (shown in [Fig. 4(a–c)]).

In [Fig. 4(a)] it can be seen that with the hybrid WO_3_ morphology, the photoanodes with and without the seed layer had comparable performance. With the WO_3_ nanoneedles there were some more notable differences. The water oxidation onset potential was cathodically shifted by ~ 150 mV with the seed layer, to ~ 0.5 V_RHE_. Despite this, the photocurrent at 1.23 V_RHE_ was similar with and without the seed layer and the plateau photocurrent was approximately 5% higher without the seed layer. This difference is attributed to changes in the morphology of the WO_3_ nanoneedles when deposited on the WO_3_ seed layer, compared to the deposition onto FTO, as illustrated in Fig. S5. The planar seed layer also had a significant effect on charge transfer. Measurements taken during water oxidation and sulfite oxidation (shown in Fig. S6), revealed that the charge injection efficiency, *η*_inj_ was enhanced with the addition of the seed layer, from 74.1 to 83.2% at 1.23 V_RHE_. However, the photoanode with WO_3_ nanoneedles without a seed layer separated photogenerated charges most efficiently, with *η*_sep_ of 50.7%, compared to 44.3% with the seed layer.

When the photoanode structures with and without the seed layer were tested at elevated electrolyte temperatures (40°C), enhanced water oxidation performance was observed [Fig. 4(b)]. This was most prevalent in the photoanodes without the planar seed layer, which saw a slight cathodic shift in the onset potential and a 23.1% increase in photocurrent during chronoamperometry at 1.23 V_RHE_, to 2.68 mA cm^−2^. The overall improvement of water oxidation kinetics on bismuth vanadate with temperature is in qualitative agreement with other studies, though this does not necessarily reflect the mechanical stability of the photoelectrode, with degradation reported to accelerate with increasing temperatures.^[17,18]^ However, at 40°C the presence of the seed layer appeared to lower the photocurrent for both hybrid and nanostructured morphologies, suggesting a negative effect of temperature on charge separation.

Chronoamperometric stability testing (shown in [Fig. 4(c)] at 1.23 V_RHE_, with flowing electrolyte heated to 40°C showed that the photoanode structures with the planar seed layer appeared to be more mechanically robust. Although the photocurrent declined during the harsher conditions of experiments at elevated temperatures, the rate of degradation was inhibited with the use of a seed layer. Whereas delamination of the photocatalyst occurred on over one third of the active surface of the electrodes without a seed layer, less than 20% of the active surface was removed from the electrodes with the seed layer. Figure 4(d, e) shows SEM images in [Fig. 4(d, e)] show that nanoneedles grown on the planar seed layer appeared to have very good adhesion to the flat WO_3_ and were less easily mechanically damaged compared to those grown directly on FTO. Conversely, the nanoneedles grown directly on FTO were mechanically degraded across the whole surface of the electrode, in agreement with previous work, the nanoneedles grown on the WO_3_ seed layer broadly retained their “as-synthesized” morphology.^[10]^ As shown in [Fig. 4(e)], the photocatalyst, while appearing relatively unchanged in terms of morphology across over 80% of the electrode surface, was completely stripped from the FTO in the remaining active area. In contrast, in the electrodes without the seed layer, remnants of damaged nanoneedles remained on FTO surface. Additional SEM images captured after the stability tests are provided in Fig S7. The results of performance and stability testing of the photoanode structures with and without the planar WO_3_ seed layer are summarized in Table S2.

The relative adhesion of the photoanodes with and without the planar WO_3_ seed layer can be explained in terms of crystallographic matching and physical anchoring. This is illustrated in [Fig. 4(f, g)]. As evidenced in Fig. 3, the WO_3_ layers (both planar and nanoneedles) have monoclinic crystal structures, whereas the FTO layer has a tetragonal structure. As a result of the poor lattice matching between FTO and WO_3_, there is a weak bond at this interface. The planar WO_3_ seed layer helps to mitigate the weak bonding between FTO and WO_3_. During the CVD reaction, the planar seed layer grows at the FTO surface, resulting in strong physical anchoring at the FTO | WO_3_ interface. In contrast, the WO_3_ nanoneedles begin to grow in the vapor phase before depositing on the surface of the FTO, leading to weaker physical anchoring. This subsequently results in the very poor adhesion of the WO_3_ nanoneedles and the good adhesion of the planar WO_3_. As planar and nanoneedle WO_3_ have monoclinic lattices, there is a strong bond at this interface. Similarly, WO_3_ and BiVO_4_ both have monoclinic structures, meaning that the WO_3_ and BiVO_4_ phases bond strongly together. The primary driver of the adhesion of the photoanode is therefore the interactions at the FTO | WO_3_ interface. This also explains the appearance of the electrodes with and without the seed layer observed by post hoc electron microscopy.

Scalability and reproducibility are two additional aspects critical to ensure that photoelectrodes can be taken to the device testing stage and integrated with up-scaled photoelectrochemical reactors. Our AA-CVD method is low-cost and scalable, enabling the fabrication of large format electrodes. In a typical batch, 12 photoelectrodes with dimensions 2.5 cm × 1.5 cm (i.e., total area ~ 45 cm^2^) can be prepared with homogeneous coatings and reproducible performances. A photograph of a representative batch of WO_3_ | BiVO_4_ | NiFeOOH photoanodes is provided in Fig S8. To validate the reproducibility of the synthesis method, the water oxidation performance of ten WO_3_ | BiVO_4_ | NiFeOOH photoanodes was characterized by linear sweep voltammetry (shown in Fig S9). These results show that this method can be translated to prepare larger, singular electrodes beyond the 1 cm^2^ scale and this will be the subject of future work.

## Discussion

Although it is most practicable for up-scaled photoelectrochemical systems to operate continuously, with flow circuitry to enable simultaneous reactant delivery to the photoelectrode surface and product harvesting, few studies have conducted stability studies with flowing electrolytes, or indeed recognized the importance of photoelectrode mechanical stability. Highly crystalline, well-ordered photoanode structures with improved mechanical stability can not only be advantageous in terms of photoelectrochemical performance, but can also significantly decrease degradation in BiVO_4_-based electrodes, even in quiescent systems, as a result of the shear stress exerted on the photocatalyst surface by evolving or accumulating gas bubbles.^[5,10,19,20]^

Although BiVO_4_-based electrodes are one of the most well-studied photoanode materials in the literature, they are inherently unstable at typical photoanode operating potentials (i.e., > 1.0 V_RHE_) according to thermodynamic predictions.^[21]^ Stability studies have shown that BiVO_4_ degradation manifests in two mechanisms: The chemical leaching of V^5+^ ions and the oxidation of Bi^3+^ ions.^[7,8]^ These mechanisms can be observed by ICP-MS electrolyte analysis showing the presence of bismuth and vanadium ions in the electrolyte during and after photoelectrochemical measurements.^[8]^ Previous studies have shown that bismuth ions are prone to oxidation in solution, forming bismuth-rich BiO_x_ on the surface of the photoanode.^[8,22]^ One of the most efficacious strategies to mitigate both mechanisms simultaneously is through the integration of water oxidation surface co-catalysts. Co-catalysts address V^5+^ leaching by providing a partial barrier at the BiVO_4_|electrolyte interface and limit Bi^3+^ oxidation by providing more active sites for photogenerated holes to oxidize water.^[8,10,23]^ WO_3_ | BiVO_4_ | NiFeOOH electrodes have been previously optimized to deliver stable photoelectrochemical water oxidation performance in pH 9 sodium borate buffer in a flow cell, with an electrolyte velocity of approximately 0.5 cm s^−1^, although mechanical and photoelectrochemical degradation was reported at higher electrolyte velocities.^[10]^ Due to the conformal growth of subsequent photoanode layers on the WO_3_ layer, which interfaces with the FTO, the adhesion and mechanical stability of this layer directly correlates to that of the whole photoanode system. The use of nanostructuring is an effective strategy to overcome issues that are inherent in metal oxide-based photoanode systems, including short minority carrier diffusion lengths and low absorption coefficients. Nanostructured photoelectrodes can absorb light and transfer charge more efficiently, resulting in greatly enhanced photocurrents compared with planar electrodes.^[5]^ In previous work by the authors, nanoneedle-based WO_3_ electrodes saw a more than fivefold increase in current density compared with planar WO_3_.^[10]^

The performance benefits of nanostructured WO_3_ must be traded-off with its poor adhesion and mechanical robustness. While planar WO_3_, also grown by CVD, has been demonstrated to adhere well to FTO and withstand flow-induced shear stress, nanostructured WO_3_ delaminates under the action of a flowing electrolyte.^[9,10]^ The use of a “hybrid” structure of WO_3_ has previously been shown to suppress the mechanical degradation of WO_3_ | BiVO_4_ | NiFeOOH photoanodes in a flow cell, but these materials still experienced some degradation under high electrolyte flowrates.^[10]^

To ensure that direct solar water splitting by photoelectrochemistry is as resilient to the challenges presented by scale-up, it is necessary to go further, to ensure the robustness of the photoelectrodes in the wide range of operating conditions that systems may operate in. A few studies have considered the effect of temperature on BiVO_4_-based photoanodes, although this is mostly limited to the effect of temperature on photoelectrochemical performance. With electrolyte temperature likely affected by the ambient conditions of outdoor operation in up-scaled photoelectrochemical systems, considering its effect on robustness of the materials is critical to ensure long-term stable operation.^[5,20,24]^

## Conclusions

This study investigated the effects of morphology and temperature on the performance and stability of WO_3_ | BiVO_4_ | NiFeOOH for photoelectrochemical water oxidation under conditions, which are relevant to future industrial deployment. To improve the mechanical stability of the WO_3_ | BiVO_4_ | NiFeOOH photoanode system in more severe operating conditions, a planar seed layer of WO_3_ on FTO, on which the WO_3_ nanoneedles were grown was integrated into the photoanode structure. As well as improving the mechanical durability, photoanodes with the seed layer exhibited improved photoelectrochemical performance. In quiescent room temperature electrolyte, the photocurrent density at 1.23 V_RHE_ was enhanced to 2.10 mA cm^−2^ from 1.85 mA cm^−2^ with the addition of the seed layer. Additionally, the onset potential was shifted cathodically by 150 mV, to 0.50 V_RHE_ and the charge injection efficiency increased to 83.2%.

To investigate the mechanisms of photoanode degradation, chronoamperometry testing (1.23 V_RHE_, one sun irradiance) at elevated electrolyte temperatures (40°C) and moderate electrolyte velocity (1.5 cm s^−1^) was carried out on nanostructured electrodes with and without the planar seed layer. This electrolyte temperature and flow velocity were selected to replicate operating conditions experienced in our up-scaled water splitting demonstration device under irradiation by natural sunlight. With the seed layer, the photocurrent during stability testing declined more slowly and had a higher photoelectrochemical performance in post hoc testing. In comparison to photoanodes without the seed layer, which were severely degraded over their entire surface, post hoc microscopy after 5-h chronoamperometry testing showed that the morphology of the photocatalyst remained relatively unchanged, with the layers well-adhered to each other. Without the seed layer, the nanostructured materials were very fragile and were rapidly damaged in the continuously flowing cell, resulting in the reduction in photocurrent, even before the photocatalyst delaminated from the FTO. The primary mechanism of photoanode degradation in the electrodes with the seed layer was the eventual, total delamination of the photocatalyst from the FTO, which appeared to be driven by the combination of the high temperature of the substrate and flow-induced shear stress while operating at an electrode potential of 1.23 V_RHE_. Due to operation with elevated electrolyte temperatures, the temperature gradient between the electrolyte and the electrode was insufficient to cool the photoanode adequately, resulting in the delamination of the photocatalyst. Temperature induced photoelectrode degradation is underexplored and with heat extraction from the photoelectrodes a key benefit of photoelectrochemical systems, this finding highlights a new challenge which should be investigated in future work.

This work provides insight into the challenges of developing stable, scalable and highly active photoelectrode materials, and the need to replicate more extreme operating conditions to test the limitations of photoanode systems. Herein, we have demonstrated the efficacy of using a planar seed layer to improve both the mechanical robustness and photoelectrochemical performance of the WO_3_ | BiVO_4_ | NiFeOOH photoanode system, establishing a foundation for further seed layer engineering to enhance the long-term stability of photoanode architectures for sustainable solar fuels production.

## Supplementary Information

Below is the link to the electronic supplementary material.

Supplementary file1 (DOCX 17521 KB)

## Data Availability

Data reported in this manuscript are available via figshare at 10.6084/m9.figshare.29626247.
