# Supplementary material for: Investigating the beneficial effects of a WO3 seed layer on the mechanical and photoelectrochemical stability of WO3|BiVO4|NiFeOOH photoanodes under operational conditions
Source: MRS Commun. 2025 Aug 18;15(4):721–30. doi: 10.1557/s43579-025-00788-9 (PMC12484363; doi:10.1557/s43579-025-00788-9)
Supplement: Supplementary file 1 — Supplementary file1 (DOCX 17521 KB) [file 43579_2025_788_MOESM1_ESM.docx]

# Supplementary information

**Investigating the beneficial effects of a WO_3_ seed layer on the mechanical and photoelectrochemical stability of WO_3_ | BiVO_4_ | NiFeOOH photoanodes under operational conditions**

George Creasey, Andreas Kafizas and Anna Hankin^*^

George Creasey Department of Chemical Engineering, Imperial College London, South Kensington, London, SW7 2AZ, United Kingdom

<https://orcid.org/0009-0002-9327-3253>

Andreas Kafizas Department of Chemistry, The Molecular Science Research Hub, Imperial College, London, White City, London, W12 0BZ, United Kingdom

<https://orcid.org/0000-0002-2282-4639>

Anna Hankin Department of Chemical Engineering, Imperial College London, South Kensington, London, SW7 2AZ, United Kingdom

<https://orcid.org/0000-0001-8358-2349>

* anna.hankin@imperial.ac.uk

The aerosol assisted chemical vapour deposition (AA-CVD) reactions were carried out in a custom-made reactor, shown in **Figure S1**. The CVD reactor temperature was measured using RS PRO Type K Mineral Insulated Thermocouples, which were placed inside the semicircular graphitic carbon block (Olmec). The carbon block was heated with a WATLOW G10A32-L12 heating rod (750 W, Sensemaster) and the desired temperature of the reaction was controlled by a temperature control unit (Eurotherm and Tempatron controller), which communicated with the heating rod and thermocouples.


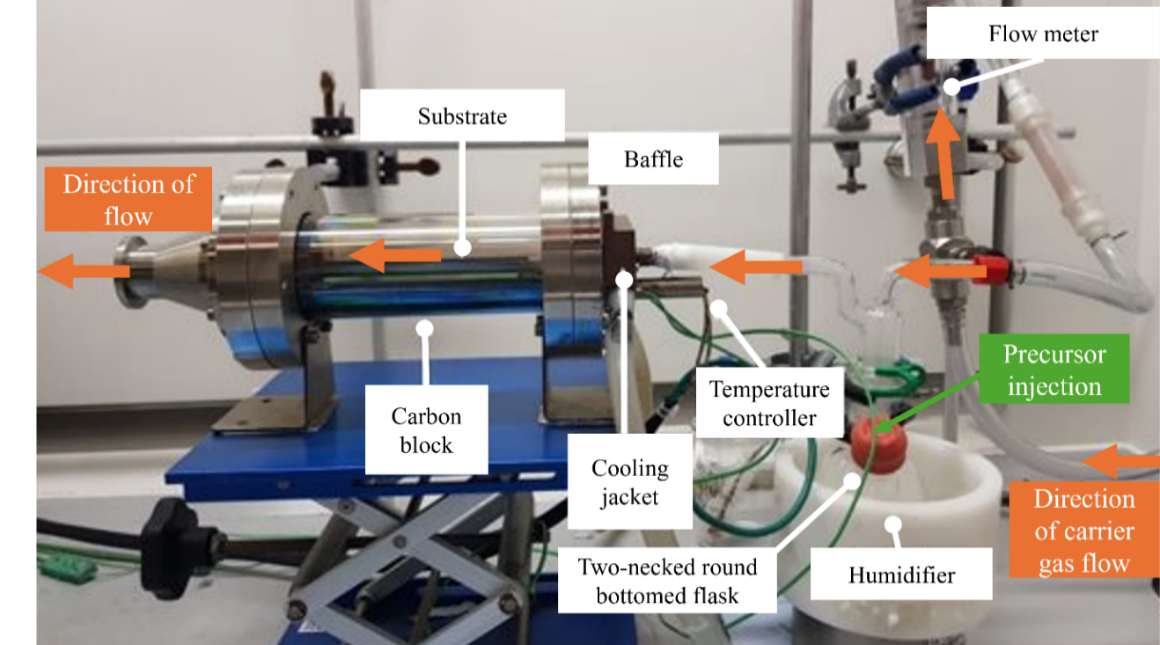


**Figure S1.** Schematic of the AA-CVD reactor used for the synthesis of WO_3_ | BiVO_4_ | NiFeOOH photoanodes. Adapted under the terms of the Creative Commons BY 3.0 licence ^10^. Copyright 2025, The Royal Society of Chemistry.

The photoelectrochemical cell was irradiated by a Class A Sun2000 Solar Simulator (Abet Technologies). A representative spectrum of the solar simulator, compared with the AM 1.5 G spectrum is shown in **Figure S2**. The working distance was adjusted to ensure an equivalent cumulative light intensity to one sun (100 mW cm^-2^).


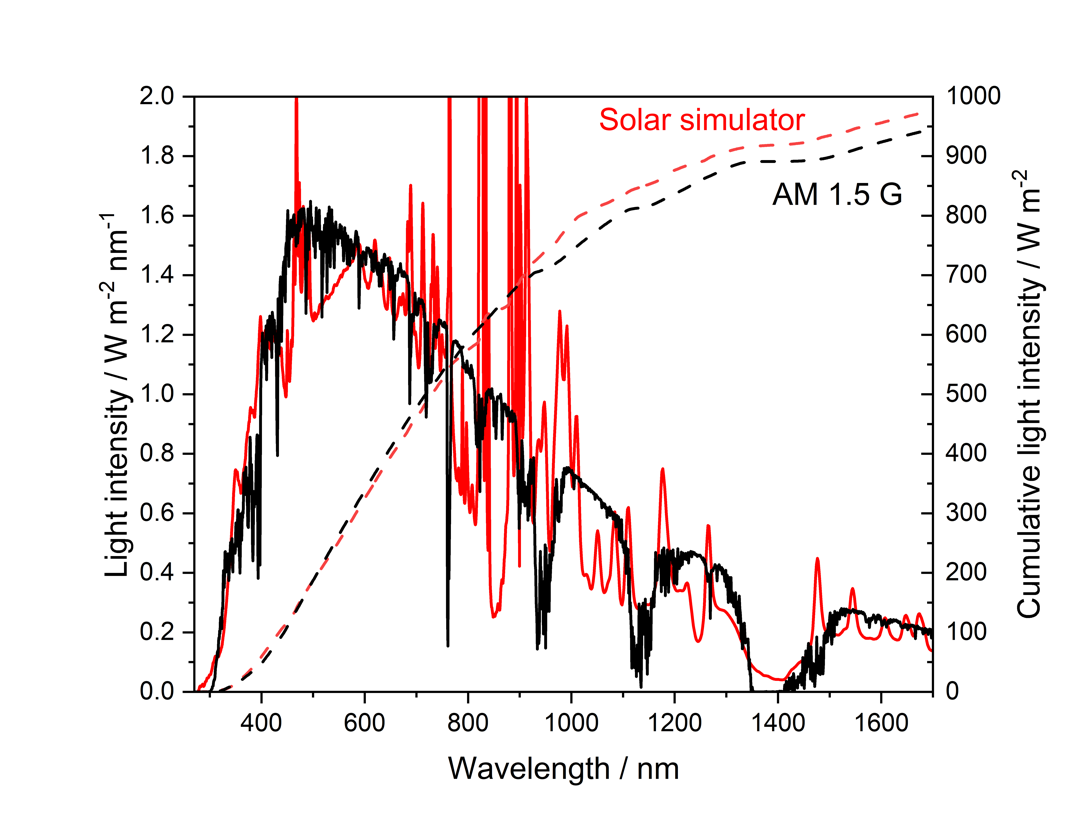


**Figure S2.** Representative spectrum of the Sun2000 Solar Simulator (Abet Technologies) used for photoelectrochemical measurements, compared with the NREL AM 1.5 G reference spectrum ^20^. Adapted under the terms of the Creative Commons BY 3.0 licence ^10^. Copyright 2025, The Royal Society of Chemistry.

**Table S1** summarises the synthesis conditions of WO_3_ in the various WO_3_ | BiVO_4_ | NiFeOOH photoanodes tested in this study. All photoanodes were prepared with the same BiVO_4_ and NiFeOOH layers sequentially deposited on WO_3_, as described in the Materials and Methods.

**Table S1.** AA-CVD synthesis conditions of WO_3_ for the various WO_3_ | BiVO_4_ | NiFeOOH photoanodes tested in this study. Photographs reproduced under the terms of the Creative Commons BY 3.0 licence ^10^. Copyright 2025, The Royal Society of Chemistry.

|  | **CVD reactor temperature (°C)** | **Nitrogen carrier gas flowrate (L min^-1^)** | **Photograph of WO_3_ \| BiVO_4_ \| NiFeOOH photoanode** |
| --- | --- | --- | --- |
| **Planar**  (and seed layers) | 325 | 1.5 | **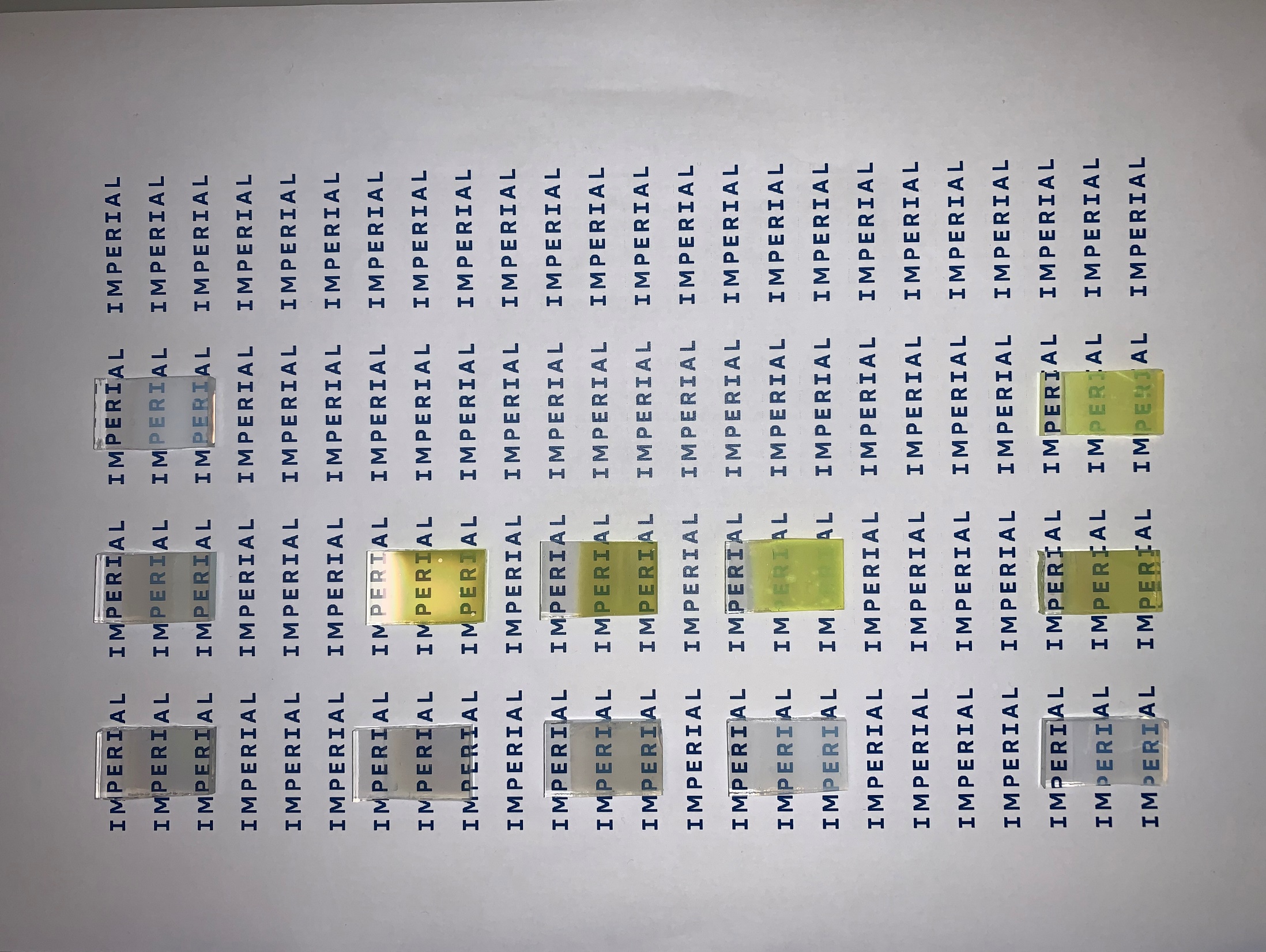** |
| **Hybrid** | 350 | 1.5 | **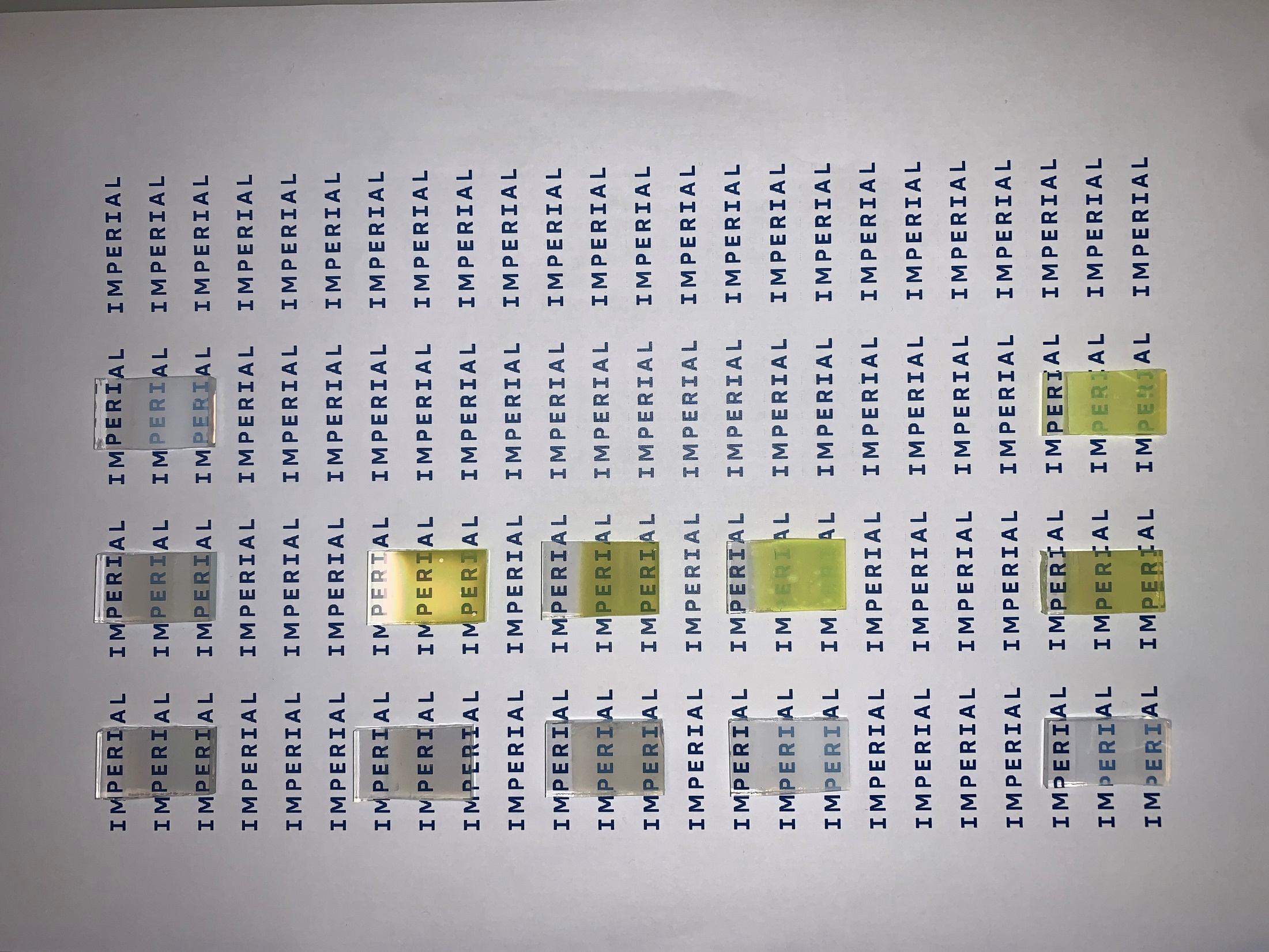** |
| **Nanoneedles** | 375 | 2.0 | **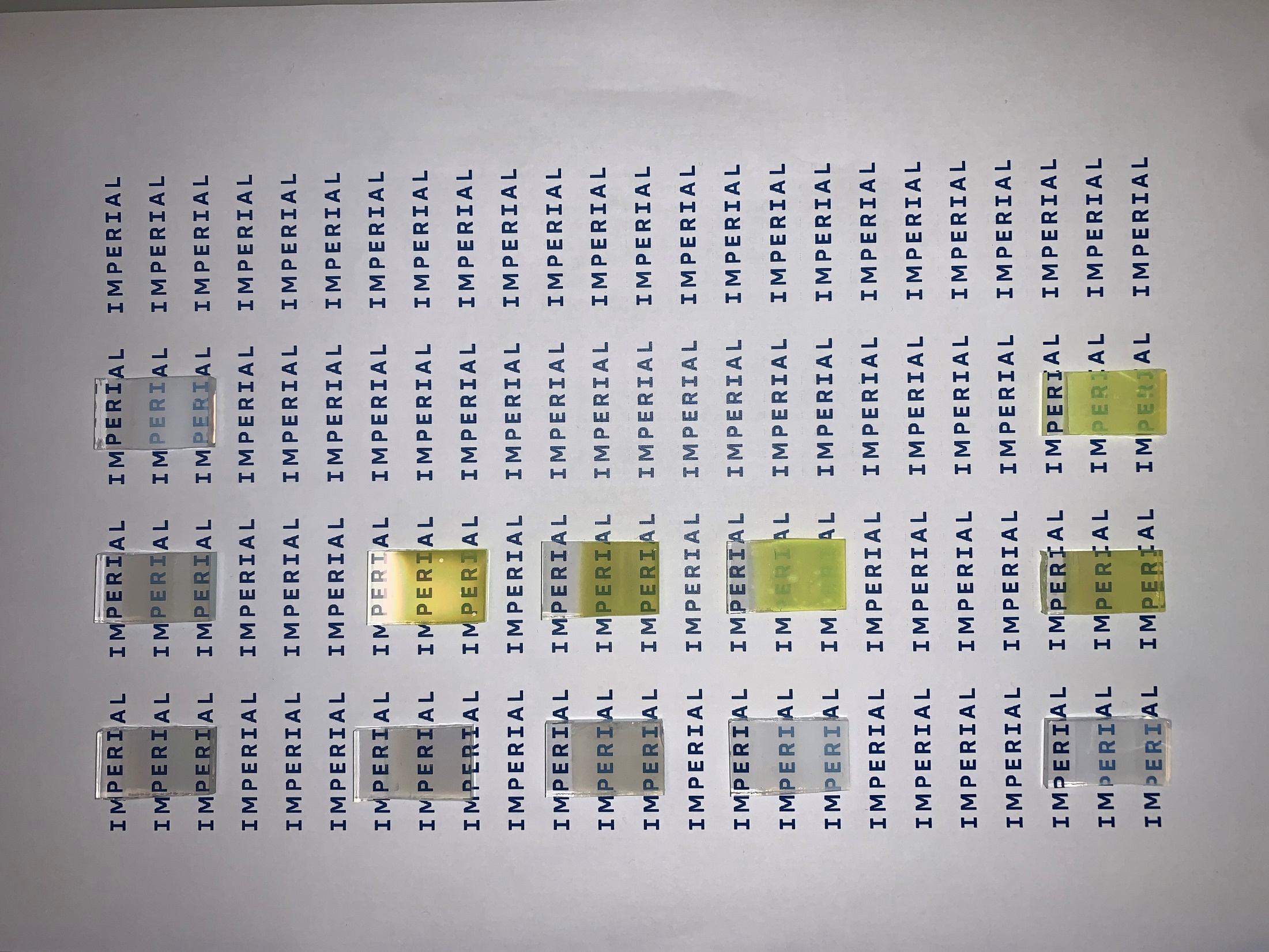** |

XPS analysis of a representative WO_3_ | BiVO_4_ | NiFeOOH sample synthesised using the same AA-CVD fabrication method was carried out previously by the authors, providing evidence of the fabrication of pure monoclinic WO_3_ (γ-WO_3_) and BiVO_4_ (m-BiVO_4_) ^10^. Analysis of the Ni 2p and Fe 2p environments also confirmed the successful loading of NiFeOOH. These results are summarised in **Figure S3** and **Figure S4**.


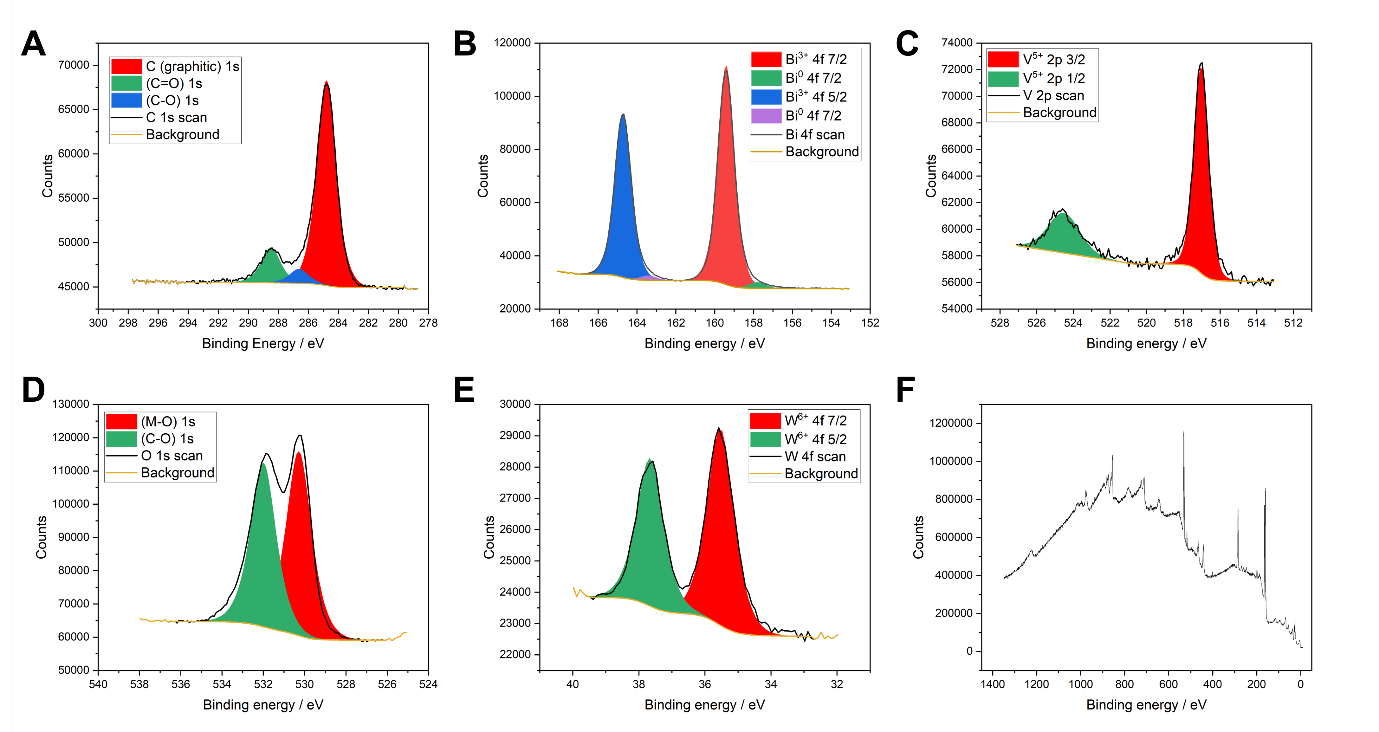


**Figure S3.** XPS analysis of a representative WO_3_ | BiVO_4_ | NiFeOOH sample deposited on FTO in the C 1s (A), Bi 4f (B), V 2p (C), O 1s (D), W 4f (E) environments and XPS survey (F). Reproduced under the terms of the Creative Commons BY 3.0 licence ^10^. Copyright 2025, The Royal Society of Chemistry.


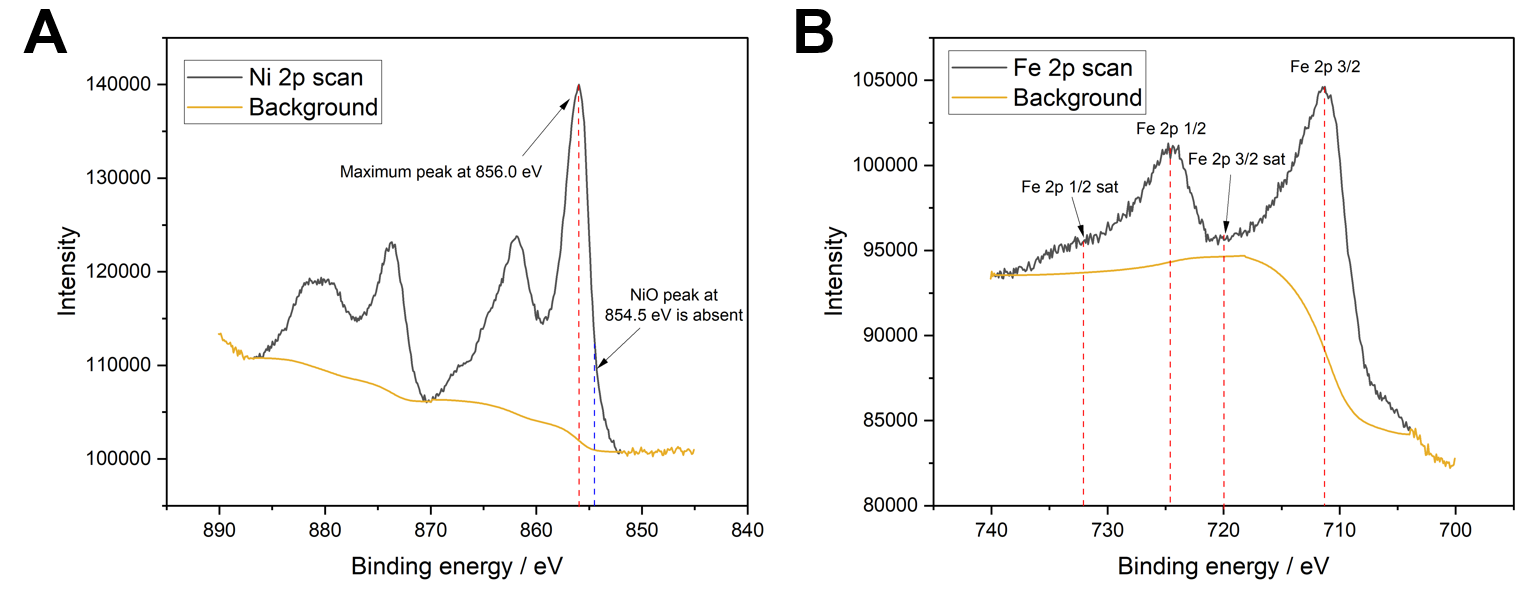


**Figure S4.** XPS analysis of a representative WO_3_ | BiVO_4_ | NiFeOOH sample deposited on FTO in the Ni 2p (A) and Fe 2p (B) environments. Reproduced under the terms of the Creative Commons BY 3.0 licence ^10^. Copyright 2025, The Royal Society of Chemistry.

**Figure S5.** SEM images of WO_3_ | BiVO_4_ | NiFeOOH electrodes. (A) WO_3_ nanoneedles with a planar WO_3_ seed layer. (B) WO_3_ nanoneedles without a planar WO_3_ seed layer.


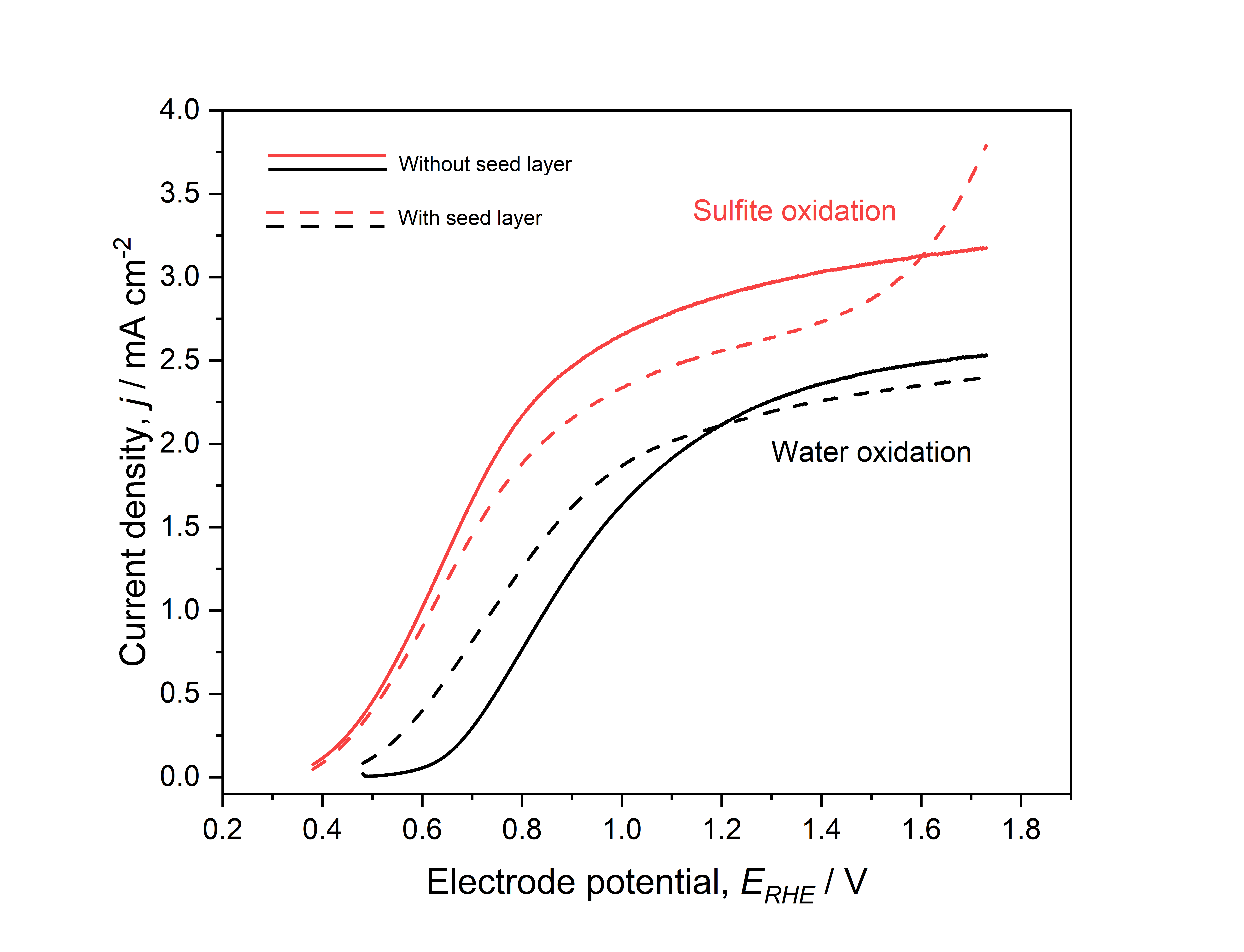


**Figure S6.** Linear sweep voltammetry measurements of WO_3_ (nanoneedles) | BiVO_4_ | NiFeOOH with and without a planar WO_3_ seed layer. Measurements were taken at room temperature in a quiescent electrolyte, with a scan rate of 10 mV s^-1^. For water oxidation, a 1 M borate buffer (pH 9) electrolyte was used, while for sulfite oxidation 0.5 M Na_2_SO_3_ was added. All measurements were conducted with one sun irradiance (100 mW cm^-2^).

**Figure S7.** SEM images (and zoomed in regions shown with arrows) of nanostructured WO_3_ | BiVO_4_ | NiFeOOH after 5-hour chronoamperometry stability tests in 100 mL min^-1^ electrolyte (1 M borate buffer, 40 °C) flow at 1.23 V_RHE_. (A) Without a planar WO_3_ seed layer. (B) With a planar WO_3_ seed layer.

**Table S2.** Summary of the water oxidation performance and stability of WO_3_ (nanoneedles) | BiVO_4_ | NiFeOOH photoanodes with and without a planar WO_3_ seed layer.

|  | **Without a seed layer** | **With a seed layer** |
| --- | --- | --- |
| **Charge injection efficiency, *η*_inj_** | 74.1% | 83.2% |
| **Charge separation efficiency, *η_sep_*** | 50.7% | 44.3% |
| **Onset potential** | ≈0.65 V_RHE_ | ≈0.50 V_RHE_ |
| **Photocurrent density at 1.23 V_RHE_ at 25°C** | 2.17 mA cm^-2^ | 2.13 mA cm^-2^ |
| **Photocurrent density at 1.23 V_RHE_ at 40°C** | 2.68 mA cm^-2^ | 2.46 mA cm^-2^ |
| **5 hour stability test at 1.23 V_RHE_ at 40°C with an electrolyte flow velocity of 1.25 cm s^-1^** | Photocurrent fell to 28% of its initial value.  Photocatalyst delaminates from the FTO with some remnants of WO_3_ nanoneedles left of the FTO surface. Photoanode mechanically degraded over entire surface. | Photocurrent fell to 44% of its initial value.  Photocatalyst delaminates completely from the FTO. In regions where delamination does not occur, WO_3_ nanoneedles remain adhered to WO_3_ seed layer with unchanged morphology. |


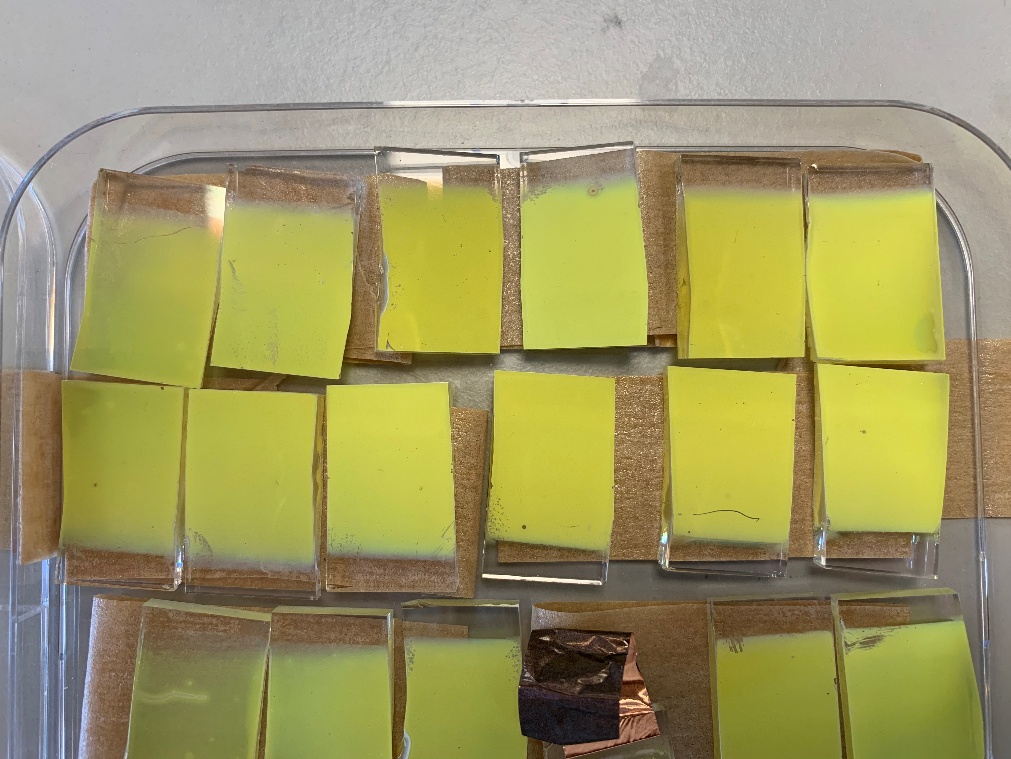


**Figure S8.** Example of a typical batch of WO_3_ (nanoneedles with seed layer) | BiVO_4_ | NiFeOOH electrodes, prepared by AA-CVD. The approximate dimension of each electrode is 1.5 cm × 2.5 cm.


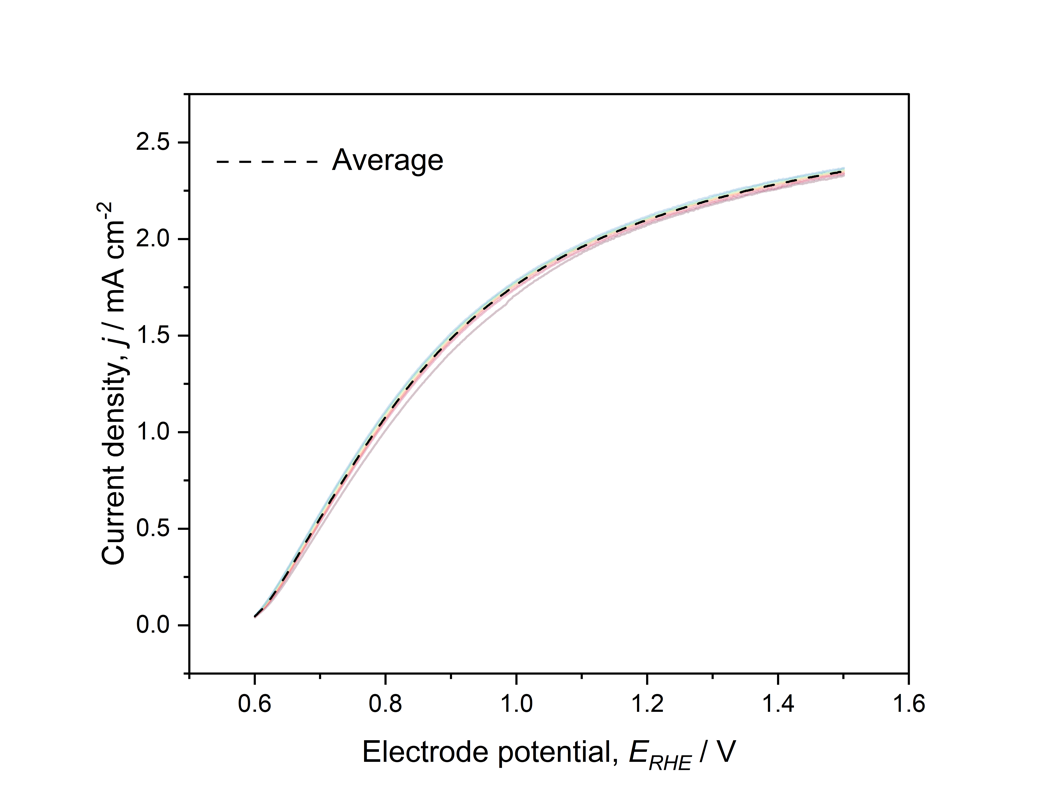


**Figure S9.** Linear sweep voltammetry of ten WO_3_ (nanoneedles) | BiVO_4_ | NiFeOOH electrodes prepared by the same AA-CVD method (in accordance with the procedures described in the methodology for synthesising WO_3_ nanoneedles, BiVO_4_ and NiFeOOH layers). Measurements were taken at room temperature in a quiescent cell, with a scan rate of 10 mV s^-1^. All measurements were conducted with one sun irradiance (100 mW cm^-2^) in a 1 M borate buffer electrolyte (pH 9).
